# The safety and clinical effectiveness of rapid infusion with CT‐P10 in patients with non‐Hodgkin's lymphoma or chronic lymphocytic leukemia: A retrospective non‐interventional post‐authorization safety study in Europe

**DOI:** 10.1002/hon.2978

**Published:** 2022-03-17

**Authors:** Mark Bishton, Scott Marshall, Jatinder Harchowal, Gilles Salles, Camille Golfier, Alessandra Tucci, Alicia Rodriguez Fernández, Jose Javier Sanchez Blanco, Monica Bocchia, SooKyoung Kim, Young Nam Lee, Pier Luigi Zinzani

**Affiliations:** ^1^ Nottingham City Hospital Nottingham University Hospitals NHS Trust Nottingham UK; ^2^ Sunderland Royal Hospital South Tyneside and Sunderland NHS Foundation Trust Sunderland UK; ^3^ The Royal Marsden NHS Foundation Trust London UK; ^4^ Centre Hospitalier Lyon Sud ‐ Service d’Hématologie Clinique Lyon France; ^5^ Hematology Department ASST Spedali Civili di Brescia Brescia Italy; ^6^ Hematology Department Hospital Universitario Virgen de la Macarena Seville Spain; ^7^ Hematology Department Hospital Morales Meseguer de Murcia Murcia Spain; ^8^ U.O.C Ematologia, Azienda Ospedaliero‐Universitaria Senese ‐ Policlinico Santa Maria alle Scotte Siena Italy; ^9^ Celltrion Healthcare Co. Ltd. Incheon Republic of Korea; ^10^ IRCCS Azienda Ospedaliero‐Universitaria di Bologna, Istituto di Ematologia “Seràgnoli” Bologna Italy; ^11^ Dipartimento di Medicina Specialistica e Sperimentale Università di Bologna Bologna Italy

**Keywords:** biosimilar pharmaceuticals, chronic, infusion, intravenous, lymphocytic leukemia, lymphoma, non‐Hodgkin, rituximab

## Abstract

Rapid infusion (RI) of the rituximab biosimilar CT‐P10 is currently only an approved treatment regimen for the treatment of rheumatoid arthritis. Although both CT‐P10 and reference rituximab are known to be frequently administered using a RI regimen (≤90 min) in clinical practice, published data on the safety of RI of CT‐P10 in patients with NHL and CLL are limited. Hence, this study collected real‐world safety and effectiveness data on RI‐CT‐P10 from the medical records of 196 patients with NHL or CLL in 10 European centers, 6 months after the date of the first RI (index date); the infusion‐related reaction (IRR) rate was compared to previously published data. Ten percent (95% confidence interval 6%–15%; *n* = 20/196) of patients experienced an infusion‐related reaction (IRR) on day 1–2 post‐index, which was not significantly different (*p = *0.45) to the IRR rate for rituximab described in a previous meta‐analysis (8.8%). During the observation period, 2% of patients experienced grade 3–5 IRRs and 85% (*n* = 166) experienced an adverse event (non‐IRR). The most common reason for discontinuation of first‐line CT‐P10 was planned treatment completion (81%; *n* = 158). Complete response and partial response to CT‐P10 was observed in 74% (*n* = 142/192) and 22% (*n* = 42/192) of patients, respectively. The results of this real‐world study demonstrate that the safety and effectiveness profile of RI‐CT‐P10 is similar to RI of reference rituximab and therefore support the current use of RI‐CT‐P10 in patients with NHL and CLL.

## 1 INTRODUCTION

CT‐P10, licensed as Truxima^®^ in Europe, is a monoclonal antibody rituximab biosimilar approved by the European Medicines Agency,^1^ for the treatment of diseases including non‐Hodgkin's lymphoma (NHL) and chronic lymphocytic leukemia (CLL).^2^ Evidence from studies of patients with rheumatoid arthritis provided a foundation for clinical similarity between CT‐P10 and reference rituximab.^2^, ^3^, ^4^, ^5^, ^6^, ^7^ Pivotal studies were conducted in advanced, symptomatic follicular lymphoma (FL) and low tumor burden FL (LTBFL), which; demonstrated that CT‐P10 produced a similar clinical response, efficacy and safety profile to reference rituximab.^8^, ^9^, ^10^

The safety profile of rituximab is well established, with infusion‐related reactions (IRRs) are the most frequently reported adverse reaction,^11^, ^12^ which are usually mild to moderate.^13^ To minimize the risk of IRRs, the classical administration protocol for rituximab takes ∼3–4 h for the first infusion, with subsequent infusions lasting 2–3 h^14^ This infusion protocol places a significant burden on the healthcare system. Consequently, when licensing permits, health care practitioners (HCPs) frequently administer rituximab as a rapid infusion (≤90 min) for second or subsequent infusions. A survey of United Kingdom (UK) cancer centers found the use of rapid rituximab infusion protocols to be widespread.^15^ Currently, rapid infusion of European Union (EU)‐sourced rituximab, MabThera^®^ (Roche) and EU approved Truxima® (Celltrion) is only permitted in patients with rheumatoid arthritis.^1^, ^13^ However, rapid infusion of United States of America (USA)‐sourced rituximab, Rituxan® (Genentech) and US‐licensed Truxima® (Celltrion) is permitted in patients with previously untreated FL and diffuse large B‐cell lymphoma (DLBCL).^1^, ^16^, ^17^ Studies conducted to evaluate the safety of rapid infusion of rituximab in patients with hematological malignancies have shown rapid administration to be well tolerated, with low incidences of IRRs.^12^, ^18^, ^19^, ^20^, ^21^

With the exception of an abstract from a single UK center,^22^ no data are available on the safety of rapid infusion of CT‐P10. Hence, more information is needed to inform evidence‐based decisions for the prescription of CT‐P10 by HCPs. The aim of this study was to address this evidence gap by collecting real‐world data on the safety and effectiveness of rapid infusion of CT‐P10 in patients with NHL and CLL in Europe. In addition, the IRR rate obtained (primary endpoint) was compared to the IRR rates of rituximab published previously.

## 2 METHODS

### 2.1 Study design and study setting

This study was a multi‐center, retrospective, non‐interventional, post‐authorization safety study conducted in 10 European specialist treatment centers or hospitals (UK, 4; Spain, 2; France, 1 and Italy, 3) which routinely use CT‐P10 for the treatment of NHL and CLL.

In the pre‐index observation period, patient‐level data for patients with NHL or CLL from hospital medical records (including infusion records) were collected from the date of diagnosis until the index date (date of first rapid infusion of CT‐P10 [for the second or a subsequent treatment cycle]), to acquire demographic and clinical characteristics data. Safety and clinical outcome data were collected in the 6‐month post‐index observation period.

### 2.2 Participants

Patients were included if they had a confirmed diagnosis of NHL (FL or DLBCL) or CLL, had received rapidly‐infused CT‐P10 for their second or a subsequent treatment cycle (rapid = 90 min or less with a window of up to 100 min), were aged ≥18 years at date of NHL/CLL diagnosis and written informed consent was provided, where required according to local regulations in the participating country.

Patients receiving reference rituximab for any previous treatment cycles within the same line of treatment and those whose medical records were unavailable, were excluded.

### 2.3 Patient consent and local authorization

Approval was sought from institutional review boards and/or independent ethics committees and local hospitals, as appropriate for each country. In accordance with French regulations, where patient consent is not required for some non‐interventional investigations, a ‘non‐opposition’ model was used. Patients, or their next of kin were sent information about the study by post and data collection proceeded if no objections were raised within 10 working days; consent was obtained for all living patients from the UK, Spain and Italy and deceased patients' data were collected by the direct care team to preserve confidentiality.

### 2.4 Endpoints and objectives

The primary endpoint was the proportion of patients who experienced an IRR (any event from a pre‐defined list) on Day 1 or Day 2 after the index event. Secondary endpoints included a description of baseline demographics, clinical and disease characteristics and assessment of the CT‐P10 safety profile, clinical effectiveness and treatment patterns in the 6 months post‐index.

### 2.5 Statistical analyses

For the primary endpoint, the proportion of patients experiencing IRRs on Day 1 or Day 2 following the index event was calculated with 95% confidence intervals (95% CIs), in both the overall sample and patients with NHL only. These IRR rates were compared to previously published IRR rates for reference rituximab (Polwart et al. 2017^23^ [vs. overall sample] and Dakhil et al. 2014^18^ [vs. patients with NHL]) using a binomial exact test.

Secondary clinical effectiveness endpoints were stratified overall and by diagnosis (DLBCL, FL or CLL) and were assessed using information documented in the patients' medical records. The proportion of patients assessed as having a best response of complete response, partial response, stable disease or progressive disease in the 6 months post‐index was calculated. For overall survival (OS) and progression‐free survival (PFS), the proportion of patients surviving and free from disease progression at 6 months following the index date were calculated; the Kaplan‐Meier method for OS and PFS was used, calculated from index with surviving patients being censored at 6 months or the date of the last known contact.

Demographic, clinical and disease characteristics data were described using summary statistics, as appropriate to the data distribution.

Treatment patterns for NHL or CLL in patients who received rapid infusion of CT‐P10 were described for the entire study period. Summary statistics were calculated for the treatment dose and treatment duration, and a distribution for the treatment discontinuation reason. Treatment duration was analyzed using the Kaplan‐Meier method, calculated from the index event date until the date of discontinuation, with the patients that remained on treatment censored at 6 months post‐index date or the date of last infusion (if the patient was lost to follow‐up).

## 3 RESULTS

### 3.1 Patient demographics and clinical characteristics

Patient demographics and clinical characteristics are summarized in Tables 1 and 2 respectively. The study included 196 patients diagnosed with CLL (18% [*n* = 35/196]), DLBCL (58% [*n* = 114/196]) or FL (24% [*n* = 47/196]), with a median age of 67.0 (interquartile range [IQR] 58.0–74.0) years (63% [*n* = 123/196] male patients); median duration of disease prior to index was 0.2 (IQR 0.1–2.0) years. Most patients had a Charlson Comorbidity Index score, at index of 0 (58% [*n* = 113/194], missing [*n* = 2]).

**TABLE 1:** Patient demographics

Patient demographics	Overall (*n* = 196)
Age at index date (years), median (IQR)	67.0 (58.0–74.0)
Male, *n* (%)	123 (63%)
Charlson comorbidity index score at index date, *n* (% of 194)	
0	113 (58%)
1	39 (20%)
2	22 (11%)
3	15 (8%)
4	2 (1%)
5	2 (1%)
8	1 (1%)
Missing	2
Comorbidities^a^ recorded in patients, *n* (% of 196)	
Diabetes	30 (15%)
Liver disease	8 (4%)
Unrelated malignancy	17 (9%)
AIDS/HIV	0 (0%)
Cerebrovascular disease	5 (3%)
CPD	10 (5%)
Congestive heart failure	8 (4%)
Dementia	0 (0%)
Hemiplegia/paraplegia	0 (0%)
Metastatic solid tumor	1 (1%)
Myocardial infarction	6 (3%)
Peptic ulcer disease	1 (1%)
PVD	9 (5%)
Renal disease	8 (4%)
Rheumatologic disease	14 (7%)
No comorbidities	113 (58%)

Abbreviations: AIDS/HIV, acquired immunodeficiency syndrome/human immunodeficiency virus; CPD, chronic pulmonary disease; IQR, interquartile range; PVD, peripheral vascular disease.

^a^ Not mutually exclusive; categories as per Charlson Comorbidity Index.

**TABLE 2:** Clinical characteristics

Patient clinical characteristics	Overall (*n* = 196)	CLL (*n* = 35)	DLBCL (*n* = 114)	FL (*n* = 47)
Distribution of patient diagnosis	‐	35 (18%)	114 (58%)	47 (24%)
Duration of disease (years from diagnosis to index date), median (IQR)	0.2 (0.1–2.0)	‐	‐	‐
Distribution of disease duration (years), *n* (%)	*n* (% of 196)	*n* (% of 35)	*n* (% of 114)	*n* (% of 114)
<1	143 (73%)	8 (23%)	103 (90%)	32 (68%)
1 < 2	4 (2%)	1 (3%)	2 (2%)	1 (2%)
2 < 3	10 (5%)	5 (14%)	1 (1%)	4 (9%)
3 < 4	7 (4%)	5 (14%	2 (2%)	0 (0%)
4 < 5	5 (3%)	1 (3%)	1 (1%)	3 (6%)
≥5	27 (14%)	15 (43%)	5 (4%)	7 (15%)
NHL Ann Arbor stage at index date			*n* (% of 71)	*n* (% of 31)
I	‐	‐	11 (15%)	1 (3%)
II			8 (11%)	6 (19%
III			8 (11%)	6 (19%)
IV			43 (61%)	18 (58%)
Other^a^			1 (1%)	0 (0%)
Missing^b^			43	16
CLL stage (Binet) at index date		*n* (% of 25)		
A	‐	9 (36%)	‐	‐
B		7 (28%)		
C		9 (36%)		
Missing		10		
IPI score for patients with DLBCL at index			*n* (% of 82)	
0	‐	‐	6 (7%)	‐
1			15 (18%)	
2			25 (30%)	
3			26 (32%)	
4			6 (7%)	
5			4 (5%)	
Missing			32	
FLIPI score summary for patients with FL at index				*n* (% of 21)
0	‐	‐	‐	1 (5%)
1				6 (29%)
2				8 (38%)
3				6 (29%)
Missing				26

Abbreviations: CLL, chronic lymphocytic leukemia; DLBCL, diffuse large B‐cell lymphoma; FL, follicular lymphoma; FLIPI, Follicular Lymphoma International Prognostic Index; IPI, International Prognostic Index; IQR, interquartile range.

^a^ “Other” stage recorded was “1E” (*n* = 1).

^b^ Missing data also includes Lugano in some instances.

### 3.2 Primary endpoint

In the overall sample, the proportion of patients experiencing IRRs on Day 1 or Day 2 post‐index event was 10.2% (95% CI 6%–15%) (Table 3) with a total of 28 IRRs recorded in 20 patients. In patients with NHL only, 9.9% (95% CI 5%–17%) of patients experienced IRRs.

**TABLE 3:** IRRs, AEs and SAEs associated with rapid CT‐P10 infusion

IRR or AEs associated with rapid CT‐P10 infusion	Overall (*n* = 196)	CLL (*n* = 35)	DLBCL (*n* = 114)	FL (*n* = 47)
Proportion of patients experiencing any IRRs on Day 1 or Day 2 post‐index event	n (% of 196 [95% CI])	% (*n* = 35)	% (*n* = 114)	% (*n* = 47)
Index IRR	20 (10.2% [6%–15%])	4 (11%)	13 (11%)	3 (6%)
No IRR at index	176 (90% [85%–94%])	31 (89%)	101 (89%)	44 (94%)
Description of IRRs at index	% (*n* = 20)	‐	‐	‐
Fatigue	7 (35%)			
Nausea	6 (30%)			
Vomiting	3 (15%)			
Peripheral edema	2 (10%)			
Rash	2 (10%)			
Hot flush	1 (5%)			
Headache	1 (5%)			
Oropharyngeal pain	1 (5%)			
Diarrhea	1 (5%)			
Pruritus	1 (5%)			
Pyrexia	1 (5%)			
Asthenia	1 (5%)			
Erythema	1 (5%)			
IRRs by grade at index	% (*n* = 25^a^, IRRs)	‐	‐	‐
Grade 1 – Mild	20 (80%)			
Grade 2 – Moderate	4 (16%)			
Grade 3 – Severe	1 (4%)			
Missing	3			
IRR experienced at index or post‐index infusions	% (*n* = 878, infusions)	‐	‐	‐
IRR	62 (7%)			
No IRR	816 (93%)			
**Grade 3–5 IRRs experienced at index**	**% (n = 196)**			
**Experienced a Grade 3–5 IRR at index**	**1 (1%)**	**‐**	**‐**	**‐**
		
IRR relatedness (at index and post‐index) to rapid CT‐P10 infusion	n (% of 83^b^, IRRs	**‐**	**‐**	**‐**
Possibly related	14 (17%)			
Unlikely to be related	18 (22%)			
Not related	24 (29%)			
Missing	27 (33%)			
Proportion of patients experiencing AEs at index or post‐index	*n* (% of 196)	*n* (% of 35)	*n* (% of 114)	*n* (% of 47)
AEs experienced	166 (85%)	27 (77%)	99 (87%)	40 (85%)
No AEs experienced	30 (15%)	8 (23%)	15 (13%)	7 (15%)
**Proportion of patients experiencing an AE at index or post‐index by Grade (AE Grade not mutually exclusive)**	***n* (% of 196)**	***n* (% of 35)**	***n* (% of 35)**	***n* (% of 47)**
**Grade 1 AE**	**134 (68%)**	**17 (49%)**	**81 (71%)**	**36 (77%)**
**Grade 2 AE**	**108 (55%)**	**13 (37%)**	**72 (63%)**	**23 (49%)**
**Grade 3 AE**	**61 (31%)**	**9 (26%)**	**39 (34%)**	**13 (28%)**
**Grade 4 AE**	**22 (11%)**	**3 (9%)**	**13 (11%)**	**6 (13%)**
**Grade 5 AE**	**3 (2%)**	**0 (0%)**	**3 (3%)**	**0 (0%)**
**AE of unknown Grade**	**16 (8%)**	**6 (17%)**	**5 (4%)**	**5 (11%)**
**Any Grade 3–5 AE experienced**	**69 (35%)**	**12 (34%)**	**42 (37%)**	**15 (32%)**
**AE relatedness to rapid CT‐P10 infusion**	***n* (% of 892** ^c^ **, AEs)**			
**Definitely related**	**4 (0.4%)**			
**Probably related**	**8 (1%)**			
**Possibly related**	**48 (5%)**			
**Unlikely to be related**	**179 (20%)**			
**Not related**	**365 (41%)**			
**Missing**	**288 (32%)**			
Proportion of patients experiencing an SAE at index or post‐index				
SAE experienced	58 (30%	5 (14%)	5 (14%)	14 (30%)
No SAE experienced	138 (70%)	138 (70%	75 (66%)	33 (70%)

Abbreviations: AE, adverse event; CLL, chronic lymphocytic leukaemia; DLBCL, diffuse large B‐cell lymphoma; FL, follicular lymphoma; IRR, infusion‐related reaction; SAE, serious adverse event.

^a^ Patients with index IRRs, *n* = 20; IRRs overall, *n* = 28.

^b^ CT‐P10 infusions at index or post‐index associated with an IRR (*n* = 61); total IRRs at index or post‐index (*n* = 83).

^c^ Total number of AEs at index or post‐index (*n* = 892).

[Correction added on 20‐April‐2022, after original publication: In Table 3, the CLL value of ‘No AEs experienced’ was changed from 30 (15%) to 8 (23%).]

### 3.3 IRRs and adverse events (AEs) during the observation period

IRR and AE data are summarized in Table 3; additional IRR data are summarized in Table S1. The most common IRR reported at index was fatigue (35% [*n* = 7/20] of patients with IRRs) and most IRRs reported at index were mild (80% [*n* = 20/25, missing *n* = 3] of IRRs with grade recorded). One patient experienced a Grade 3 IRR at index (oropharyngeal pain, Grade 3), which was considered unlikely to be related to CT‐P10 by the investigator.

In the observation period (index and up to 6 months post‐index) there were 83 IRRs, of which 14 were considered possibly related to CT‐P10, 18 were unlikely to be related and 24 were not related (data missing for 27 IRRs). **Overall, 85% (*n* = 166/196) of patients experienced one or more AEs (any grade, excluding IRRs),** 35% (*n* = 69/196) experienced a Grade 3–5 AE and 30% (*n* = 58/196) experienced a serious AE (SAE). Of all AEs experienced at index or post‐index (*n* = 892), 12 were recorded as definitely or probably related to CT‐P10 (relatedness data missing for 288 AEs). Complete Medical Dictionary for Regulatory Activities coding of the IRR and AE data can be seen in Tables S2 and S3, respectively.

### 3.4 Comparison of observed IRR rate to published reference rituximab IRR rates

**FIGURE 1:**
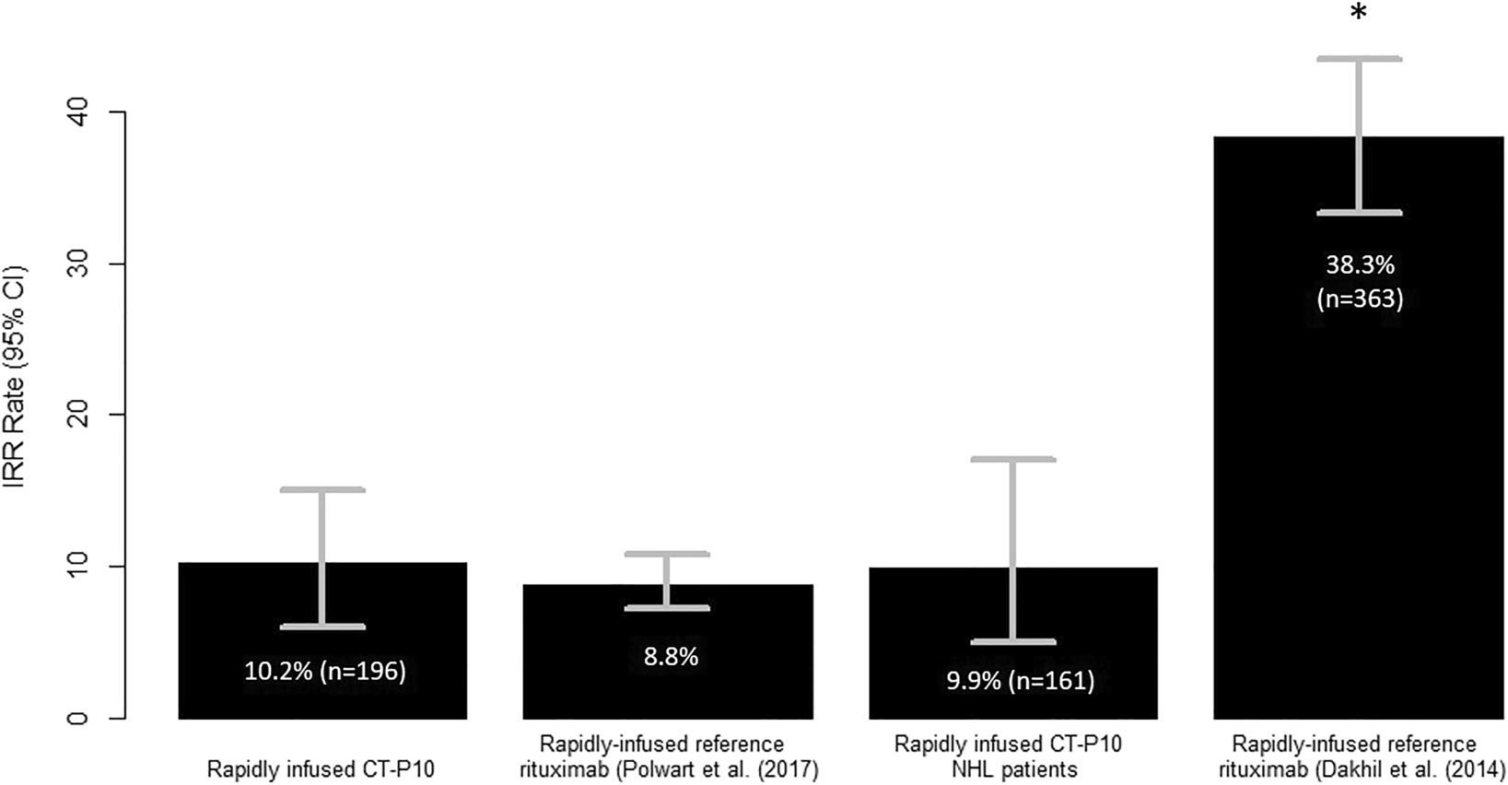
Comparison of observed IRR rate with published reference rituximab IRR rates. The observed reference rate was compared with other published IRR rates relating to rapid‐infused reference rituximab (Dakhil et al. [2014] and Polwart et al. [2017]). * indicates significance where *p* < 0.001 with a binomial exact text compared to the observed IRR rate in patients with NHL in this study; ns = not significant compared to the observed IRR rate in this study in the overall sample. IRR, infusion‐related reaction

The IRR rates from the Dakhil et al. (2014)^18^ and Polwart et al. (2017)^23^ studies were 38.3% (*n* = 139/363 [95% CI 33.3%–43.5%]) and 8.8% (95% CI 7.2%–10.8%) respectively (Figure 1). The IRR rate for CT‐P10 in the overall sample was not significantly different from the IRR rate reported in the Polwart et al. (2017)^23^ study (*p* = 0.45), but the IRR rate in patients with NHL was significantly lower than that described in the Dakhil et al. (2014)^18^ study (*p* < 0.001).

### 3.5 Treatment patterns

Treatment pattern data are summarized in Table S4. Eighty‐two percent (*n* = 160/195; this wasn't evaluable for one of the 196 patients) of patients received CT‐P10 as a first‐line treatment. Patients received a mean of 4.5 (standard deviation [SD] 1.5) CT‐P10 infusions during the observation period (index and post‐index). The median duration of index CT‐P10 infusions, where data were available, was 90.0 (IQR 90.0–90.0, *n* = 118) minutes and 90.0 (IQR, 90.0–90.0, *n* = 406) minutes for post‐index (not including index) infusions. At the end of the observation period 81% (*n* = 158/196) of patients had discontinued first‐line CT‐P10 treatment due to planned completion of the treatment course, 7% (*n* = 13/196) due to AEs, 2% (*n* = 4/196) due to disease progression, 4% (*n* = 7/196) due to other reasons and 7% (*n* = 13/196) were ongoing with the first line of treatment (*n* = 1 not known). Only 16% (*n* = 32/196) of patients received a subsequent treatment following discontinuation of CT‐P10 during the observation period. The median index dose of CT‐P10 was 375.0 mg/m^2^ (IQR 375.0–375.0) and 97% (*n* = 190/196) of patients had no dose changes recorded during the observation period (four patients had one and two patients had two dose changes). Eighteen percent (*n* = 35/195; in 1/196 patients their prior treatment status was unknown) of patients had received prior treatment for NHL or CLL at index. The most administered prior treatment was R‐CHOP (6%, *n* = 12/195), followed by rituximab monotherapy (4%, *n* = 7/195. R‐CHOP (cyclophosphamide, doxorubicin, vincristine and prednisone plus rituximab) was the most common chemotherapy regimen used at the index infusion (47% [*n* = 93/196]).

### 3.6 Clinical effectiveness

**FIGURE 2:**
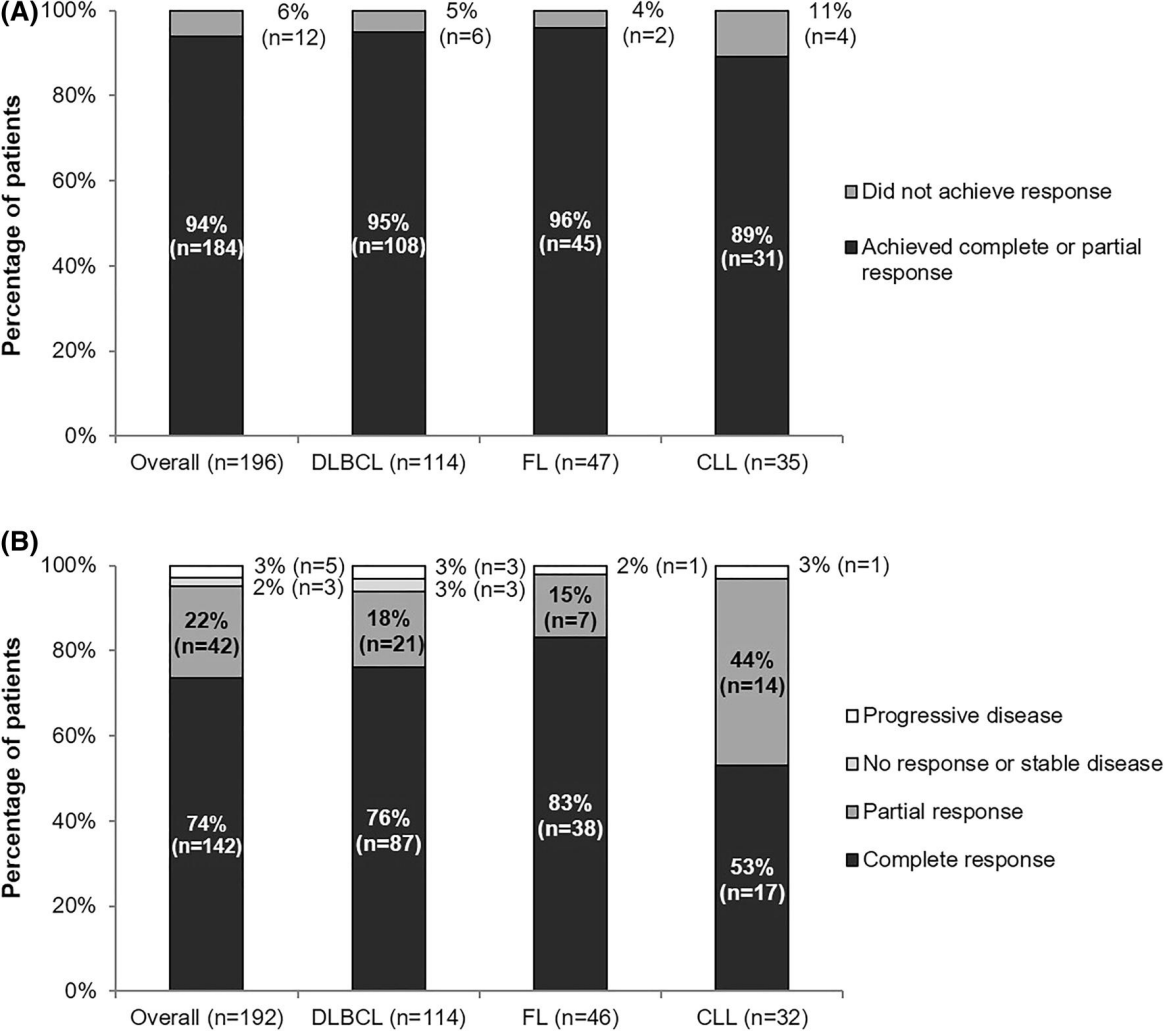
Response to CT‐P10 during the observation period. The proportion of all patients achieving a complete or partial response to CT‐P10 during the observation period is presented (A). The best response to CT‐P10 during the observation period, in patients with a response recorded, was also calculated (B) (Best response data were available for 192 patients; missing data = overall [*n* = 4]; CLL (chronic lymphocytic leukemia) [*n* = 3]; FL (follicular lymphoma) [*n* = 1]. DLBCL, diffuse large B‐cell lymphoma

Clinical effectiveness data are summarized in Figure 2. The percentage of patients achieving a complete or partial response for the overall, DLBCL, FL and CLL subgroups was 94% (*n* = 184/196), 95% (*n* = 108/114), 96% (*n* = 45/47) and 89% (*n* = 31/35), respectively (Figure 2A). Overall, the best responses were recorded as complete response (74% [*n* = 142/192]), partial response (22% [*n* = 42/192], no response or stable disease (2% [*n* = 3/192]) and progressive disease (3% [*n* = 5/192]), Figure 2B.

**FIGURE 3:**
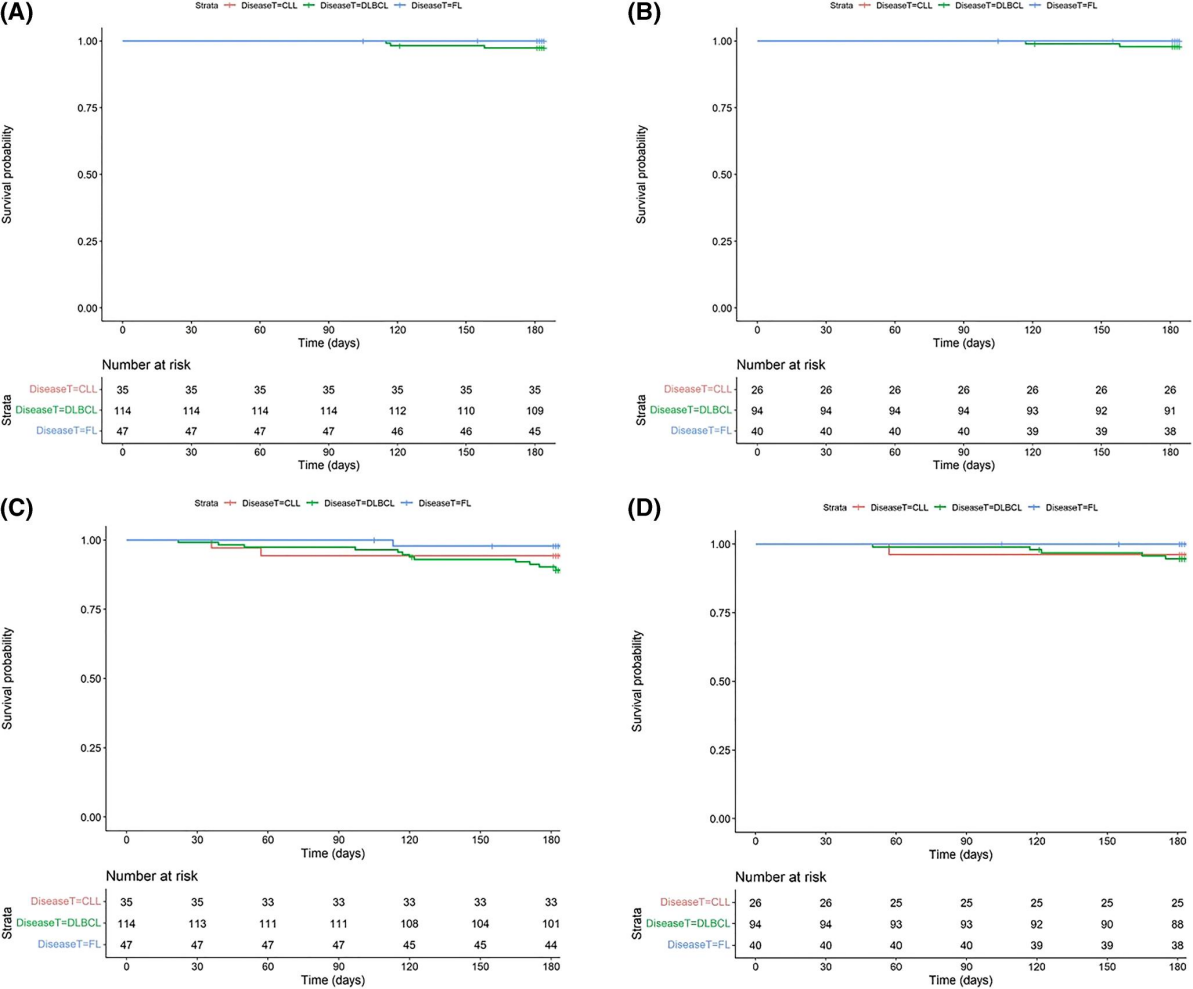
Kaplan‐Meier charts for overall survival and progression‐free survival from index for all patients and treatment‐naïve patients stratified by diagnosis. Overall survival (OS) for all patients from index stratified by diagnosis (A). OS for treatment‐naïve patients only from index stratified by diagnosis (B). Progression‐free survival (PFS) for all patients from index stratified by diagnosis (C). PFS for treatment‐naïve patients only from index stratified by diagnosis (D). CLL, chronic lymphocytic leukemia; DLBCL, diffuse large B‐cell lymphoma; FL, follicular lymphoma

OS, PFS and time to permanent CT‐P10 treatment discontinuation are summarized in Table S5. Kaplan‐Meier charts depict OS and PFS from index for all patients and treatment‐naïve patients stratified by diagnosis (Figure 3). In relation to OS, the proportion of all patients, and treatment‐naïve patients, alive at the end of the observation period was 96% (*n* = 188/196) and 96% (*n* = 154/160), respectively. Overall, the proportion of patients alive and free from disease progression at 6 months post‐index was 89% (*n* = 175/196).

CT‐P10 treatment was permanently discontinued prior to 6 months for 91% (*n* = 179/196) of patients, where the median treatment duration (Kaplan‐Meier estimate) was 89.5 (95% CI 85–99) days.

## 4 DISCUSSION

Reference rituximab and CT‐P10 are administered using an RI regimen in clinical practice, despite the lack of real‐world data relating to the safety of the RI of CT‐P10 in patients with NHL and CLL. Hence, this study collected safety and effectiveness data relating to RI of CT‐P10 and the IRR rate obtained was compared to previously published IRR rates for reference rituximab.

Two studies previously investigating rapid reference rituximab IRR rates were used to provide context to the primary endpoint data.^18^, ^23^ The first, a Phase III safety study of rituximab administered as a 90‐min infusion in patients with previously untreated DLBCL, reported an IRR rate of 38.3% following the first rapid infusion^18^ and 4 grade 3 IRRs (vs. 1 grade 3 IRR in the present study). The second was a meta‐analysis of 2472 patients conducted by Polwart et al. (2017).^23^ The meta‐analysis described an audit standard for the rapid infusion of rituximab biosimilar using data from 20 rapid rituximab infusion studies relating to hematological malignancies. An estimated IRR rate of 8.8% (95% CI 7.2%–10.8%) was reported with a suggested recommendation that the IRR rate with rituximab biosimilar should be less than 10.8% for all grades and 0.7% for grade 3 or more. In the present study (*n* = 196), the observed IRR rate of 10.2% for all grades (95% CI 6%–15%) and 0.5% for IRRs of grade 3 or more, in the overall sample falls below this threshold. Furthermore, the IRR rate was not significantly different (*p* = 0.45) from the IRR rate reported by Polwart et al. (2017) and in patients with NHL only, was significantly lower (*p* < 0.001) than the rate reported by Dakhil et al. (2014). However, the Dakhil et al. (2014) study only included treatment‐naïve patients, whereas some patients in the present study received prior lines of rituximab treatment, which could explain the difference observed in IRR rate. Differences may also be related to the ‘real‐world’ and retrospective nature of the study, whereby IRRs occurring the day after CT‐P10 infusion may be less well documented.

Previous studies demonstrate that rapid rituximab infusion administration is well‐tolerated, with low incidences of IRRs.^12^, ^18^, ^19^, ^20^, ^21^ The results of the present study support this, where 22% of patients experienced an IRR during the 6‐month observation period (Table S1), most IRRs recorded were mild or moderate and 17% (*n* = 14/83) of IRRs were considered ‘possibly related’ to CT‐P10. This is also consistent with a study assessing CT‐P10 rapid infusion safety, where the majority of IRRs were mild.^22^ In addition, similar IRR findings were reported by Shah et al. (2017), who assessed the safety of rapid infusion of Truxima in patients switching from MabThera, both with previous exposure to MabThera and rituximab‐naïve.^22^ Across all groups, the majority of IRRs were Grade 1 and only a single Grade 2/3 IRR was recorded.^22^

Among the 85% of patients who experienced AEs and 30% who experienced SAEs, most AEs were also mild or moderate. The main reason for first‐line CT‐P10 discontinuation was due to completion of the planned treatment course, as opposed to being associated with an AE. Most of the follow‐up (post‐index) infusions followed a rapid infusion protocol, which suggests that rapid infusion of CT‐P10 was generally well tolerated. For the AE profile in Dakhil et al. (2014),^18^ Grade 3/4 AEs were reported in 46% and 36.3% in the R‐CHOP and R‐CVP (rituximab plus cyclophosphamide, vincristine and prednisone) cohorts, respectively. Overall, SAEs were experienced by 19.8% of patients. In the present study, 35% of patients experienced a Grade 3–5 AE and 30% (*n* = 58/196) experienced an SAE. Therefore, these results suggest that, like reference rituximab, CT‐P10 was well tolerated in combination with chemotherapy with a safety profile is broadly similar to that reported in Dakhil et al. (2014).^18^

Traditionally, rituximab is administered over several hours with multiple infusions.^14^, ^16^ This method represents a significant financial and healthcare burden. A previous American study demonstrated that rapid infusion of rituximab in patients with DLBCL or FL resulted in direct medical savings and increased productivity for patients and HCPs.^24^ In the current study, while impact on economic costs, productivity or acceptability to patients of rapid infusion of CT‐P10 were not formally investigated, the results are supportive of the safety and effectiveness of this method of administration.

Limitations are associated with this study. For some participating countries, consent was a requirement, and may have introduced selection bias (percentage of patients from each country: UK [61%]; Spain [11%]; Italy [20%]; France [8%]). Interpretation of data collected retrospectively was dependent on the completeness of the medical records and the reliability of the abstraction of data. As highlighted, it is possible that IRRs occurring the day after the infusion were less well documented, with consequent underestimation of the overall incidence of IRRs during the observation period. The IRR rate may also be underestimated because of the definition of a rapid infusion as 90 min or less, since this may result in the exclusion of cases where the initial intention was to infuse CT‐P10 over more than 90 min, but the infusion was delayed or stopped due to IRRs. In addition, data is not available relating to patients who switched to CT‐P10 from reference rituximab within the same line of therapy. Despite the limitations, the described results will help to support treatment decisions and inform the routine clinical management of patients with CLL and NHL who receive rituximab in clinical practice.

Overall, results support the safety and effectiveness of rapid infusion of CT‐P10 in patients with NHL or CLL in the real‐world and also provides support for the current use of biosimilars, such as CT‐P10 in clinical practice.

## 5 CONCLUSION

This is the first multi‐country study to investigate the safety and effectiveness of rapid infusion of CT‐P10 in a real‐world setting. CT‐P10 was generally well tolerated, and the observed IRR rate was not significantly different from the rate reported previously in a meta‐analysis investigating the safety of reference rituximab. Overall, the results from this study demonstrate that CT‐P10 has a similar IRR rate, safety and clinical effectiveness profile to those previously reported for reference rituximab and thus provides HCPs with more detailed information on the safety profile of rapidly infused CT‐P10. This will allow informed, evidence‐based decisions on the most appropriate and cost‐effective treatment strategy for patients with CLL or NHL.

## AUTHOR CONTRIBUTIONS

SooKyoung Kim, Young Nam Lee and Pier Luigi Zinzani designed the research study. Mark Bishton, Scott Marshall, Jatinder Harchowal, Gilles Salles, Camille Golfier, Alessandra Tucci, Alicia Rodriguez Fernández, Jose Javier Sanchez Blanco, Monica Bocchia and Pier Luigi Zinzani performed the research. All authors analyzed the data and wrote the manuscript.

## CONFLICT OF INTEREST

No, there is no conflict of interest.

### PEER REVIEW

The peer review history for this article is available at https://publons.com/publon/10.1002/hon.2978.

## Supporting information

Supplementary Material

## Data Availability

The data that support the findings of this study are available from the corresponding author upon reasonable request.
