# Supplementary material for: The safety and clinical effectiveness of rapid infusion with CT‐P10 in patients with non‐Hodgkin's lymphoma or chronic lymphocytic leukemia: A retrospective non‐interventional post‐authorization safety study in Europe
Source: Hematol Oncol. 2022 Mar 17;40(3):370–80. doi: 10.1002/hon.2978 (PMC9545983; doi:10.1002/hon.2978)
Supplement: Supplementary file 1 — Supplementary Material [file HON-40-370-s001.docx]

**TABLE S1. Additional IRR data during the 6-month observation period.**

| **IRRs at index or post index** | **Overall** | | **CLL** | | **DLBCL** | | **FL** | |
| --- | --- | --- | --- | --- | --- | --- | --- | --- |
|  | **n** | **%**  **(n = 196)** | **n** | **%**  **(n = 35)** | **n** | **%**  **(n = 114)** | **n** | **%**  **(n = 47)** |
| IRR experienced at index or post-index | 44 | 22% | 7 | 20% | 29 | 25% | 8 | 17% |
| No IRR at index or post-index | 152 | 78% | 28 | 83% | 85 | 75% | 39 | 83% |
| **IRRs post-index (not including index)** |  |  |  |  |  |  |  |  |
| IRR experienced post-index | 31 | 16% | 4 | 11% | 20 | 18% | 7 | 15% |
| No IRR post-index | 165 | 84% | 31 | 89% | 94 | 82% | 40 | 85% |
| **Grade 3, 4 or 5 IRRs at index or post-index** |  |  |  |  |  |  |  |  |
| Grade 3–5 IRR experienced at index or post-index | 3 | 2% | 0 | 0% | 3 | 3% | 0 | 0% |
| No Grade 3–5 IRR at index or post-index | 193 | 98% | 35 | 100% | 111 | 97% | 47 | 100% |
| **Grade 3, 4 or 5 IRRs post-index (not including index)** |  |  |  |  |  |  |  |  |
| Grade 3–5 IRR experienced post-index | 2 | 1% | 0 | 0% | 2 | 2% | 0 | 0% |
| No Grade 3–5 IRR post-index | 194 | 99% | 35 | 100% | 112 | 98% | 47 | 100% |

CLL, chronic lymphocytic leukaemia; DLBCL, diffuse large B-cell lymphoma; FL, follicular lymphoma; IRR, infusion-related reaction.

**TABLE S2. Medical Dictionary for Regulatory activities (MedDRA) description of IRRs.**

| **Primary system organ class** | **IRRs (n = 83)** | | **Patients (n = 196)** | |
| --- | --- | --- | --- | --- |
| **Preferred term** | **n** | **(%)** | **n** | **(%)** |
| **Gastrointestinal signs and symptoms** | **30** | **36%** | **30** | **15%** |
| Nausea | 24 | 29% | 24 | 12% |
| Vomiting | 6 | 7% | 6 | 3% |
| **Vascular disorders NEC** | **1** | **1%** | **1** | **1%** |
| Hot flush | 1 | 1% | 1 | 1% |
| **General system disorders NEC** | **31** | **37%** | **31** | **16%** |
| Asthenia | 8 | 10% | 8 | 4% |
| Fatigue | 23 | 28% | 23 | 12% |
| **Electrolyte and fluid balance conditions** | **2** | **2%** | **2** | **1%** |
| Oedema | 2 | 2% | 2 | 1% |
| **Cardiac arrhythmias** | **1** | **1%** | **1** | **1%** |
| Tachycardia | 1 | 1% | 1 | 1% |
| **Epidermal and dermal conditions** | **7** | **8%** | **7** | **4%** |
| Rash | 3 | 4% | 3 | 2% |
| Pruritus | 3 | 4% | 3 | 2% |
| Erythema | 1 | 1% | 1 | 1% |
| **Headaches** | **1** | **1%** | **1** | **1%** |
| Headache | 1 | 1% | 1 | 1% |
| **Respiratory tract signs and symptoms** | **1** | **1%** | **1** | **1%** |
| Oropharyngeal pain | 1 | 1% | 1 | 1% |
| **Gastrointestinal motility and defaecation conditions** | **3** | **4%** | **3** | **2%** |
| Diarrhoea | 3 | 4% | 3 | 2% |
| **Allergic conditions** | **1** | **1%** | **1** | **1%** |
| Hypersensitivity | 1 | 1% | 1 | 1% |
| **Cardiac disorder signs and symptoms** | **1** | **1%** | **1** | **1%** |
| Dyspnoea | 1 | 1% | 1 | 1% |
| **Neurological disorders NEC** | **2** | **2%** | **2** | **1%** |
| Dizziness | 2 | 2% | 2 | 1% |
| **Upper respiratory tract disorders (excluding infections)** | **1** | **1%** | **1** | **1%** |
| Laryngeal oedema | 1 | 1% | 1 | 1% |
| **Body temperature conditions** | 1 | 1% | 1 | 1% |
| Pyrexia | 1 | 1% | 1 | 1% |

IRR, infusion-related reaction; MedDRA, Medical Dictionary for Regulatory Activities; NEC, not elsewhere classified.

**TABLE S3. MedDRA description of AEs.**

| **Primary system organ class** | **AEs (n = 892)** | | **Patients (n = 196)** | |
| --- | --- | --- | --- | --- |
| **Preferred term** | **n** | **(%)** | **n** | **(%)** |
| **Infections: pathogen unspecified** | **53** | **6%** | **53** | **27%** |
| Upper respiratory tract infection | 2 | 0% | 2 | 1% |
| Pneumonia | 3 | 0% | 3 | 2% |
| Septic shock | 1 | 0% | 1 | 1% |
| Rhinitis | 4 | 0% | 4 | 2% |
| Cystitis | 1 | 0% | 1 | 1% |
| Infective spondylitis | 1 | 0% | 1 | 1% |
| Neutropenic sepsis | 14 | 2% | 14 | 7% |
| Vascular device infection | 1 | 0% | 1 | 1% |
| Urinary tract infection | 3 | 0% | 3 | 2% |
| Pharyngitis | 1 | 0% | 1 | 1% |
| Lower respiratory tract infection | 6 | 1% | 6 | 3% |
| Folliculitis | 2 | 0% | 2 | 1% |
| Conjunctivitis | 1 | 0% | 1 | 1% |
| Sepsis | 2 | 0% | 2 | 1% |
| Oral infection | 1 | 0% | 1 | 1% |
| Infection | 2 | 0% | 2 | 1% |
| Groin infection | 1 | 0% | 1 | 1% |
| Tooth abscess | 1 | 0% | 1 | 1% |
| Tooth infection | 1 | 0% | 1 | 1% |
| Nail bed infection | 1 | 0% | 1 | 1% |
| Paronychia | 1 | 0% | 1 | 1% |
| Respiratory tract infection | 1 | 0% | 1 | 1% |
| Nasopharyngitis | 1 | 0% | 1 | 1% |
| Lung infection | 1 | 0% | 1 | 1% |
| **Hepatobiliary investigations** | **15** | **2%** | **15** | **8%** |
| Transaminases increased | 1 | 0% | 1 | 1% |
| Blood bilirubin increased | 5 | 1% | 5 | 3% |
| Alanine aminotransferase increased | 8 | 1% | 8 | 4% |
| Aspartate aminotransferase increased | 1 | 0% | 1 | 1% |
| **Gastrointestinal motility and defaecation conditions** | **47** | **5%** | **47** | **24%** |
| Diarrhoea | 27 | 3% | 27 | 14% |
| Constipation | 20 | 2% | 20 | 10% |
| **Bone and joint injuries** | **3** | **0%** | **3** | **2%** |
| Lumbar vertebral fracture | 2 | 0% | 2 | 1% |
| Spinal compression fracture | 1 | 0% | 1 | 1% |
| **White blood cell disorders** | **74** | **8%** | **74** | **38%** |
| Neutropenia | 70 | 8% | 70 | 36% |
| Febrile neutropenia | 4 | 0% | 4 | 2% |
| **Body temperature conditions** | **32** | **4%** | **32** | **16%** |
| Pyrexia | 32 | 4% | 32 | 16% |
| **Musculoskeletal and connective tissue disorders NEC** | **17** | **2%** | **17** | **9%** |
| Pain in extremity | 5 | 1% | 5 | 3% |
| Back pain | 10 | 1% | 10 | 5% |
| Musculoskeletal disorder | 1 | 0% | 1 | 1% |
| Musculoskeletal chest pain | 1 | 0% | 1 | 1% |
| **General system disorders NEC** | **89** | **10%** | **89** | **45%** |
| Asthenia | 14 | 2% | 14 | 7% |
| Mucosal inflammation | 13 | 1% | 13 | 7% |
| Fatigue | 42 | 5% | 42 | 21% |
| Extravasation | 1 | 0% | 1 | 1% |
| Peripheral swelling | 2 | 0% | 2 | 1% |
| Disease progression | 2 | 0% | 2 | 1% |
| Generalised oedema | 1 | 0% | 1 | 1% |
| Oedema peripheral | 3 | 0% | 3 | 2% |
| Chills | 4 | 0% | 4 | 2% |
| Face oedema | 1 | 0% | 1 | 1% |
| Axillary pain | 1 | 0% | 1 | 1% |
| Night sweats | 2 | 0% | 2 | 1% |
| Influenza-like illness | 2 | 0% | 2 | 1% |
| Chest pain | 1 | 0% | 1 | 1% |
| **Gastrointestinal signs and symptoms** | **61** | **7%** | **61** | **31%** |
| Nausea | 40 | 4% | 40 | 20% |
| Abdominal pain upper | 4 | 0% | 4 | 2% |
| Abdominal pain | 6 | 1% | 6 | 3% |
| Vomiting | 9 | 1% | 9 | 5% |
| Dyspepsia | 1 | 0% | 1 | 1% |
| Abnormal faeces | 1 | 0% | 1 | 1% |
| **Epidermal and dermal conditions** | **25** | **3%** | **25** | **13%** |
| Pruritus | 4 | 0% | 4 | 2% |
| Skin lesion | 1 | 0% | 1 | 1% |
| Rash | 13 | 1% | 13 | 7% |
| Dry skin | 3 | 0% | 3 | 2% |
| Neurodermatitis | 1 | 0% | 1 | 1% |
| Erythema | 3 | 0% | 3 | 2% |
| **Viral infectious disorders** | **9** | **1%** | **9** | **5%** |
| Influenza | 5 | 1% | 5 | 3% |
| Herpes zoster | 3 | 0% | 3 | 2% |
| Herpes virus infection | 1 | 0% | 1 | 1% |
| **Respiratory disorders NEC** | **18** | **2%** | **18** | **9%** |
| Cough | 18 | 2% | 18 | 9% |
| **Peripheral neuropathies** | **35** | **4%** | **35** | **18%** |
| Neuropathy peripheral | 28 | 3% | 28 | 14% |
| Peripheral sensory neuropathy | 6 | 1% | 6 | 3% |
| Carpal tunnel syndrome | 1 | 0% | 1 | 1% |
| **Bone disorders excluding congenital and fractures** | **5** | **1%** | **5** | **3%** |
| Bone pain | 5 | 1% | 5 | 3% |
| **Ocular infections, irritations and inflammations** | **2** | **0%** | **2** | **1%** |
| Eye pruritus | 1 | 0% | 1 | 1% |
| Blepharitis | 1 | 0% | 1 | 1% |
| **Upper respiratory tract disorders excluding infections** | **1** | **0%** | **1** | **1%** |
| Nasal congestion | 1 | 0% | 1 | 1% |
| **Neurological disorders NEC** | **17** | **2%** | **17** | **9%** |
| Hypoaesthesia | 1 | 0% | 1 | 1% |
| Dysgeusia | 2 | 0% | 2 | 1% |
| Paraesthesia | 4 | 0% | 4 | 2% |
| Dizziness | 6 | 1% | 6 | 3% |
| Taste disorder | 2 | 0% | 2 | 1% |
| Presyncope | 1 | 0% | 1 | 1% |
| Dysaesthesia | 1 | 0% | 1 | 1% |
| **Hepatic and hepatobiliary disorders** | **3** | **0%** | **3** | **2%** |
| Jaundice | 1 | 0% | 1 | 1% |
| Hepatocellular injury | 1 | 0% | 1 | 1% |
| Hepatic steatosis | 1 | 0% | 1 | 1% |
| **Joint disorders** | **7** | **1%** | **7** | **4%** |
| Arthralgia | 4 | 0% | 4 | 2% |
| Polyarthritis | 1 | 0% | 1 | 1% |
| Osteoarthritis | 1 | 0% | 1 | 1% |
| Temporomandibular joint syndrome | 1 | 0% | 1 | 1% |
| **Headaches** | **10** | **1%** | **10** | **5%** |
| Headache | 10 | 1% | 10 | 5% |
| **Anaemias non-haemolytic and marrow depression** | **79** | **9%** | **79** | **40%** |
| Anaemia | 75 | 8% | 75 | 38% |
| Febrile bone marrow aplasia | 3 | 0% | 3 | 2% |
| Pancytopenia | 1 | 0% | 1 | 1% |
| **Platelet disorders** | **24** | **3%** | **24** | **12%** |
| Thrombocytopenia | 23 | 3% | 23 | 12% |
| Thrombocytosis | 1 | 0% | 1 | 1% |
| **Encephalopathies** | **1** | **0%** | **1** | **1%** |
| Encephalopathy | 1 | 0% | 1 | 1% |
| **Immune disorders NEC** | **1** | **0%** | **1** | **1%** |
| Cytokine release syndrome | 1 | 0% | 1 | 1% |
| **Muscle disorders** | **5** | **1%** | **5** | **3%** |
| Muscle spasms | 4 | 0% | 4 | 2% |
| Myalgia | 1 | 0% | 1 | 1% |
| **Oral soft tissue conditions** | **31** | **3%** | **31** | **16%** |
| Odynophagia | 1 | 0% | 1 | 1% |
| Stomatitis | 16 | 2% | 16 | 8% |
| Throat irritation | 6 | 1% | 6 | 3% |
| Oral pain | 3 | 0% | 3 | 2% |
| Aphthous ulcer | 3 | 0% | 3 | 2% |
| Mouth ulceration | 2 | 0% | 2 | 1% |
| **Gastrointestinal vascular conditions** | **2** | **0%** | **2** | **1%** |
| Haemorrhoids | 2 | 0% | 2 | 1% |
| **Fungal infectious disorders** | **7** | **1%** | **7** | **4%** |
| Mucocutaneous candidiasis | 1 | 0% | 1 | 1% |
| Oral candidiasis | 6 | 1% | 6 | 3% |
| **Prostatic disorders excluding infections and inflammations** | **1** | **0%** | **1** | **1%** |
| Benign prostatic hyperplasia | 1 | 0% | 1 | 1% |
| **Male reproductive tract infections and inflammations** | **1** | **0%** | **1** | **1%** |
| Prostatitis | 1 | 0% | 1 | 1% |
| **Bacterial infectious disorders** | **7** | **1%** | **7** | **4%** |
| Bacterial prostatitis | 1 | 0% | 1 | 1% |
| *Clostridium* colitis | 1 | 0% | 1 | 1% |
| *Clostridium difficile* infection | 1 | 0% | 1 | 1% |
| *Pneumonia staphylococcal* | 1 | 0% | 1 | 1% |
| Cellulitis | 1 | 0% | 1 | 1% |
| *Klebsiella* sepsis | 2 | 0% | 2 | 1% |
| **Injuries NEC** | **5** | **1%** | **5** | **3%** |
| Dust inhalation pneumopathy | 1 | 0% | 1 | 1% |
| Limb injury | 1 | 0% | 1 | 1% |
| Road traffic accident | 1 | 0% | 1 | 1% |
| Skin laceration | 1 | 0% | 1 | 1% |
| Contusion | 1 | 0% | 1 | 1% |
| **Cardiac disorders signs and symptoms NEC** | **2** | **0%** | **2** | **1%** |
| Cyanosis | 2 | 0% | 2 | 1% |
| **Gastrointestinal inflammatory conditions** | **2** | **0%** | **2** | **1%** |
| Gastritis | 1 | 0% | 1 | 1% |
| Colitis | 1 | 0% | 1 | 1% |
| **Cardiac disorder signs and symptoms** | **11** | **1%** | **11** | **6%** |
| Dyspnoea | 9 | 1% | 9 | 5% |
| Palpitations | 2 | 0% | 2 | 1% |
| **Embolism and thrombosis** | **6** | **1%** | **6** | **3%** |
| Deep vein thrombosis | 2 | 0% | 2 | 1% |
| Subclavian vein thrombosis | 1 | 0% | 1 | 1% |
| Embolism | 1 | 0% | 1 | 1% |
| Brachiocephalic vein thrombosis | 1 | 0% | 1 | 1% |
| Thrombophlebitis | 1 | 0% | 1 | 1% |
| **Cardiac arrhythmias** | **7** | **1%** | **7** | **4%** |
| Arrhythmia | 1 | 0% | 1 | 1% |
| Bradycardia | 1 | 0% | 1 | 1% |
| Tachycardia | 3 | 0% | 3 | 2% |
| Atrial fibrillation | 1 | 0% | 1 | 1% |
| Ventricular extrasystoles | 1 | 0% | 1 | 1% |
| **Vascular haemorrhagic disorders** | **2** | **0%** | **2** | **1%** |
| Epistaxis | 2 | 0% | 2 | 1% |
| **Pleural disorders** | **4** | **0%** | **4** | **2%** |
| Pleural effusion | 4 | 0% | 4 | 2% |
| **Electrolyte and fluid balance conditions** | **13** | **1%** | **13** | **7%** |
| Oedema | 9 | 1% | 9 | 5% |
| Hypokalaemia | 4 | 0% | 4 | 2% |
| **Vasovagal symptoms** | **2** | **0%** | **2** | **1%** |
| Vasovagal symptoms | 2 | 0% | 2 | 1% |
| **Decreased and nonspecific blood pressure disorders and shock** | **7** | **1%** | **7** | **4%** |
| Hypotension | 6 | 1% | 6 | 3% |
| Syncope | 1 | 0% | 1 | 1% |
| **Urinary tract signs and symptoms** | **7** | **1%** | **7** | **4%** |
| Dysuria | 2 | 0% | 2 | 1% |
| Chromaturia | 1 | 0% | 1 | 1% |
| Pollakiuria | 2 | 0% | 2 | 1% |
| Nocturia | 2 | 0% | 2 | 1% |
| **Renal disorders excluding nephropathies** | **2** | **0%** | **2** | **1%** |
| Acute kidney injury | 2 | 0% | 2 | 1% |
| **Purine and pyrimidine metabolism disorders** | **1** | **0%** | **1** | **1%** |
| Gout | 1 | 0% | 1 | 1% |
| **Hearing disorders** | **2** | **0%** | **2** | **1%** |
| Deafness | 1 | 0% | 1 | 1% |
| Hypoacusis | 1 | 0% | 1 | 1% |
| **Neuromuscular disorders** | **2** | **0%** | **2** | **1%** |
| Autonomic neuropathy | 2 | 0% | 2 | 1% |
| **Coronary artery disorders** | **1** | **0%** | **1** | **1%** |
| Chest discomfort | 1 | 0% | 1 | 1% |
| **Deliria including confusion** | **1** | **0%** | **1** | **1%** |
| Confusional state | 1 | 0% | 1 | 1% |
| **Appetite and general nutritional disorders** | **11** | **1%** | **11** | **6%** |
| Decreased appetite | 11 | 1% | 11 | 6% |
| **Depressed mood disorders and disturbances** | **2** | **0%** | **2** | **1%** |
| Depression | 2 | 0% | 2 | 1% |
| **Gastrointestinal haemorrhages NEC** | **2** | **0%** | **2** | **1%** |
| Melaena | 1 | 0% | 1 | 1% |
| Haematochezia | 1 | 0% | 1 | 1% |
| **Physical examination and organ system status topics** | **3** | **0%** | **3** | **2%** |
| Weight decrease | 2 | 0% | 2 | 1% |
| Weight decreased | 1 | 0% | 1 | 1% |
| **Exocrine pancreas conditions** | **1** | **0%** | **1** | **1%** |
| Pancreatitis acute | 1 | 0% | 1 | 1% |
| **Anxiety disorders and symptoms** | **4** | **0%** | **4** | **2%** |
| Anxiety | 4 | 0% | 4 | 2% |
| **Vascular disorders NEC** | **1** | **0%** | **1** | **1%** |
| Hot flush | 1 | 0% | 1 | 1% |
| **Reproductive tract disorders NEC** | **2** | **0%** | **2** | **1%** |
| Haematuria | 2 | 0% | 2 | 1% |
| **Aneurysms and artery dissections** | **1** | **0%** | **1** | **1%** |
| Aortic aneurysm | 1 | 0% | 1 | 1% |
| **Fatal outcomes** | **1** | **0%** | **1** | **1%** |
| Death | 1 | 0% | 1 | 1% |
| **Skin appendage conditions** | **5** | **1%** | **5** | **3%** |
| Alopecia | 4 | 0% | 4 | 2% |
| Nail dystrophy | 1 | 0% | 1 | 1% |
| **Respiratory tract signs and symptoms** | **9** | **1%** | **9** | **5%** |
| Oropharyngeal pain | 6 | 1% | 6 | 3% |
| Rhinorrhoea | 2 | 0% | 2 | 1% |
| Dysphonia | 1 | 0% | 1 | 1% |
| **Haematology investigations including blood groups** | **44** | **5%** | **44** | **22%** |
| Neutrophil count decreased | 20 | 2% | 20 | 10% |
| Platelet count decreased | 16 | 2% | 16 | 8% |
| White blood cell count decreased | 3 | 0% | 3 | 2% |
| Platelet count increased | 1 | 0% | 1 | 1% |
| Lymphocyte count decreased | 4 | 0% | 4 | 2% |
| **Eye disorders NEC** | **3** | **0%** | **3** | **2%** |
| Dry eye | 2 | 0% | 2 | 1% |
| Eye disorder | 1 | 0% | 1 | 1% |
| **Schizophrenia and other psychotic disorders** | **1** | **0%** | **1** | **1%** |
| Substance induced psychotic disorder | 1 | 0% | 1 | 1% |
| **Testicular and epididymal disorders** | **2** | **0%** | **2** | **1%** |
| Testicular disorder | 1 | 0% | 1 | 1% |
| Testicular pain | 1 | 0% | 1 | 1% |
| **Spleen: lymphatic and reticuloendothelial system disorders** | **1** | **0%** | **1** | **1%** |
| Lymphadenopathy | 1 | 0% | 1 | 1% |
| **Renal and urinary tract investigations and urinalyses** | **2** | **0%** | **2** | **1%** |
| Blood creatinine increased | 2 | 0% | 2 | 1% |
| **Complications associated with device** | **1** | **0%** | **1** | **1%** |
| Device-related infection | 1 | 0% | 1 | 1% |
| **Enzyme investigations NEC** | **1** | **0%** | **1** | **1%** |
| Blood alkaline phosphatase increased | 1 | 0% | 1 | 1% |
| **Salivary gland conditions** | **3** | **0%** | **3** | **2%** |
| Dry mouth | 3 | 0% | 3 | 2% |
| **Aural disorders NEC** | **2** | **0%** | **2** | **1%** |
| Ear pain | 2 | 0% | 2 | 1% |
| **Ocular sensory symptoms NEC** | **1** | **0%** | **1** | **1%** |
| Foreign body sensation in eyes | 1 | 0% | 1 | 1% |
| **Inner ear and VIII^th^ cranial nerve disorders** | **1** | **0%** | **1** | **1%** |
| Tinnitus | 1 | 0% | 1 | 1% |
| **Lower respiratory tract disorders excluding obstruction and infection** | **1** | **0%** | **1** | **1%** |
| Pulmonary oedema | 1 | 0% | 1 | 1% |
| **Administration site reactions** | **5** | **1%** | **5** | **3%** |
| Injection site reaction | 1 | 0% | 1 | 1% |
| Infusion site phlebitis | 1 | 0% | 1 | 1% |
| Infusion site pain | 3 | 0% | 3 | 2% |
| **Bladder and bladder neck disorders excluding calculi** | **3** | **0%** | **3** | **2%** |
| Cystitis noninfective | 3 | 0% | 3 | 2% |
| **Cutaneous neoplasms benign** | **1** | **0%** | **1** | **1%** |
| Seborrhoeic keratosis | 1 | 0% | 1 | 1% |
| **Bile duct disorders** | **1** | **0%** | **1** | **1%** |
| Biliary colic | 1 | 0% | 1 | 1% |
| **Heart failures** | **1** | **0%** | **1** | **1%** |
| Cardiac failure | 1 | 0% | 1 | 1% |
| **Venous varices** | **1** | **0%** | **1** | **1%** |
| Varicose vein | 1 | 0% | 1 | 1% |
| **Vitamin-related disorders** | **1** | **0%** | **1** | **1%** |
| Folate deficiency | 1 | 0% | 1 | 1% |
| **Pulmonary vascular disorders** | **1** | **0%** | **1** | **1%** |
| Pulmonary embolism | 1 | 0% | 1 | 1% |
| **Soft tissue neoplasms benign** | **1** | **0%** | **1** | **1%** |
| Lipoma | 1 | 0% | 1 | 1% |
| **Procedural related injuries and complications NEC** | **2** | **0%** | **2** | **1%** |
| Infusion-related reaction | 2 | 0% | 2 | 1% |
| **Skin and subcutaneous tissue disorders NEC** | **1** | **0%** | **1** | **1%** |
| Skin mass | 1 | 0% | 1 | 1% |
| **Cardiac and vascular investigations excluding enzyme tests** | **1** | **0%** | **1** | **1%** |
| Cardiac stress test | 1 | 0% | 1 | 1% |
| **Vision disorders** | **1** | **0%** | **1** | **1%** |
| Visual impairment | 1 | 0% | 1 | 1% |
| **Vascular therapeutic procedures** | **1** | **0%** | **1** | **1%** |
| Vascular graft | 1 | 0% | 1 | 1% |
| **Anal and rectal conditions NEC** | **1** | **0%** | **1** | **1%** |
| Rectal tenesmus | 1 | 0% | 1 | 1% |
| **Allergic conditions** | **1** | **0%** | **1** | **1%** |
| Contrast media allergy | 1 | 0% | 1 | 1% |
| **Thyroid gland disorders** | **1** | **0%** | **1** | **1%** |
| Goitre | 1 | 0% | 1 | 1% |
| **Dental and gingival conditions** | **2** | **0%** | **2** | **1%** |
| Toothache | 2 | 0% | 2 | 1% |
| **Angioedema and urticaria** | **1** | **0%** | **1** | **1%** |
| Swelling face | 1 | 0% | 1 | 1% |
| **Reproductive tract and breast disorders congenital** | **1** | **0%** | **1** | **1%** |
| Hydrocoele | 1 | 0% | 1 | 1% |

AE, adverse event; MedDRA, Medical Dictionary for Regulatory Activities; NEC, not elsewhere classified.

**TABLE S4. Treatment patterns in patients with NHL or CLL who received treatment with rapidly infused CT-P10.**

| **Treatment information** | **Overall (n = 196)** |
| --- | --- |
| Number of CT-P10 infusions prior to index (mean, SD)  Distribution of infusions (n, %)  1  2  3  4  5  8 | 1.6 (1.1)  107 (55%)  71 (36%)  12 (6%)  2 (1%)  1 (1%)  3 (2%) |
| Position of CT-P10 in treatment pathway  First-line treatment  Second-line treatment  Third-line treatment  Fourth-line treatment or more  Missing | n (% of 195)  160 (82%)  19 (10%)  10 (5%)  6 (3%)  1 |
| Number of CT-P10 infusions per patient (mean, SD)  Distribution of number of infusions post-index (including index infusion) (n, %)  1  2  3  4  5  6  7  8 | 4.5 (1.5)  5 (3%)  21 (11%)  20 (10%)  44 (22%)  61 (31%)  26 (13%)  18 (9%)  1 (1%) |
| Duration (minutes) of index CT-P10 infusion for each patient, median (IQR)  Distribution of duration of index CT-P10 infusions  <60  60<90  ≥90  Missing | 90.0 (90.0–90.0)  n (% of 118)  3 (3%)  3 (3%)  112 (95%)  78 |
| Duration (minutes) of all post-index (not including index) CT-P10 infusions, median (IQR)  Missing | n = 406  90.0 (90.0–90.0)  276 |
| Reasons for discontinuation of first-line CT-P10 treatment  Completed course of treatment  AE  First line ongoing  Disease progression  Other (specify)^†^  Not known | n (% of 196)  158 (81%)  13 (7%)  13 (7%)  4 (2%)  7 (4%)  1 (1%) |
| Reasons for discontinuation of second-line of CT-P10 treatment  Completed course of treatment  Disease progression  No second line^‡^ | n (% of 196)  10 (5%)  1 (1%)  185 (94%) |
| Number and proportion of patients receiving a subsequent treatment following discontinuation of CT-P10 during the observation period  Yes  No | n (% of 196)  10 (5%)  1 (1%)  185 (94%) |
| Number and proportion of patients with recorded prior treatments for NHL or CLL at the index date  Prior treatment  No prior treatment  Missing | n (% of 195)  35 (18%)  160 (82%)  1 |
| Prior treatments recorded  R-CHOP  Rituximab monotherapy  R-CVP  FCR  Cyclophosphamide  Chlorambucil and rituximab  Idelalisib and rituximab  Other therapy^§^  No prior treatment | n (% of 195)  12 (6%)  7 (4%)  2 (1%)  3 (2%)  3 (2%)  3 (2%)  1 (1%)  18 (9%)  160 (82%) |
| Chemotherapy regimen used at index infusion  B-rituximab  Chlorambucil-rituximab  DA-EPOCH-rituximab  FC-rituximab  R-CHOP  R-CVP  R-GCVP  R-IVAC  R-mini-CHOP  Rituximab monotherapy  Other (specify)^¶^ | n (% of 196)  44 (22%)  1 (1%)  1 (1%)  14 (7%)  93 (47%)  3 (2%)  4 (2%)  2 (1%)  2 (1%)  4 (2%)  28 (14%) |
| AE, adverse event; CLL, chronic lymphocytic leukaemia; FCR, fludarabine, cyclophosphamide and rituximab; IQR, interquartile range; NHL, non-Hodgkin’s lymphoma; R-CHOP, rituximab plus cyclophosphamide, doxorubicin hydrochloride, vincristine and prednisolone; R-CVP, rituximab plus cyclophosphamide, vincristine sulphate and prednisolone; SD, standard deviation. Rituximab = reference rituximab.  ^†^ “Other (specify)” reasons for discontinuation were, “Risk of toxicities” (n = 2), “General poor health” (n = 1), “Multidisciplinary team decision due to complete response post cycle three” (n = 1), “Myelodysplastic syndrome” (n = 1), “Refractory disease” (n = 1) and “Surgery” (n = 1).  ^‡^This indicates that the patient had not yet initiated a second line of treatment during the observation period.  ^§^Other therapies included: BR (bendamustine [treanda] and rituximab); chlorambucil-obinutuzumab; obinutuzumab; methotrexate; R-ESHAP (rituximab, etoposide, solu-medrone, high-dose cytarabine and cisplatin); R-ICE (rituximab, ifosfamide, carboplatin and etoposide); R-CHOP–Myocet; Polatuzumab Vedotin; R-GDP (rituximab, gemcitabine, dexamethasone and cisplatin); MATRix (methotrexate, ara-C [cytarabine], thiotepa and rituximab), prednisolone; R-IEV (rituximab, ifosfamide, epirubicin, etoposide); fludarabine and cyclophosphamide; fludarabine and mitoxantrone; rituximab and bortezomib; Node Exeresis; CHOP; R-CHP (rituximab, cyclophosphamide, doxorubicin and prednisone); chlorambucil, rituximab and idelalisib; R-CNOP (rituximab-cyclophosphamide, mitoxantrone, vincristine and prednisone).  ^¶^Other (specify) = chlorambucil-rituximab (n = 1); MATRix (methotrexate, ara-C [cytarabine], thiotepa and rituximab) (n = 2); PMitCEBO-rituximab (prednisolone, mitoxantrone, cyclophosphamide, etoposide, vincristine, and bleomycin-rituximab) (n = 1); R-ACVBP (rituximab, doxorubicin, cyclophosphamide, vindesine, bleomycin, prednisolone) (n = 2); R-CHOEP (rituximab, cyclophosphamide, doxorubicin vincristine, etoposide and prednisolone) (n = 1); R-CHOP and IT (intrathecal) methotrexate (n = 4); R-COMP (prednisone, cyclophosphamide, vincristine, Myocet^TM^ and rituximab) (n = 1); R-CVP (n = 1); R-DHAOx (n = 2); R-GEMOX (n = 3); R-Holoxan VP16 (n = 1); R-idealisib (n = 2); R-MACOP-B (rituximab, methotrexate, doxorubicin, cyclophosphamide, vincristine, prednisone, and bleomycin) (n = 1); R-PCEGEMBO (rituximab-cyclophosphamide, etoposide, gemcitabine, vincristine, bleomycin) (n = 1); R-PCEGemBOM (rituximab-cyclophosphamide, etoposide, gemcitabine, vincristine, methotrexate and bleomycin) (n = 1); R-CHOP+ intravenous high-dose methotrexate central nervous system prophylaxis (n = 1); R-GDP and IT methotrexate (n = 1); rituximab – folic acid maintenance (n = 1); rituximab and COMP (50% total dose), (n = 1). | |

**TABLE S5. Survival, disease progression and time to final CT-P10 treatment discontinuation.**

| **Survival, disease progression and time to final CT-P10 discontinuation** | **Overall**  **(n = 196)** | **CLL**  **(n = 35)** | **DLBCL**  **(n = 114)** | **FL**  **(n = 47)** |
| --- | --- | --- | --- | --- |
| Proportion of patients alive at the end of the observation period  Alive  Deceased  Not known  Proportion of treatment naïve patients alive at the end of the observation period  Alive  Deceased  Not known  Proportion of patients alive and free from disease progression at the end of the observation period  Progressed or died  Alive and progression free  Not known | n (% of 196)  188 (96%)  3 (2%)  5 (3%)  n (% of 160)  154 (96%)  2 (1%)  4 (2%)  n (% of 196)  16 (8%)  175 (89%)  5 (3%) | n (% of35)  35 (100%)  0  0  n (% of 26)  26 (100%)  0  0  n (% of 35)  2 (6%)  33 (94%)  0 | n (% of 114)  108 (95%)  3 (3%)  3 (3%)  n (% of 94)  90 (96%)  2 (2%)  2 (2%)  n (% of 114)  13 (11%)  98 (86%)  3 (3%) | n (% of 47)  45 (96%)  0  2 (4%)  n (% of 40)  38 (95%)  0  2 (5%)  n (% of 47)  1 (2%)  44 (94%)  2 (4%) |
| CT-P10 discontinued during follow-up?  Discontinued prior to 6 months  Ongoing treatment (censored)  Treatment duration (days)  Median (95% CI); Kaplan-Meier estimate | n (% of 196)  179 (91%)  17 (9%)  89.5  (85–99) | n (% of 35)  29 (83%)  6 (17%)  113.0  (109–134) | n (% of 114)  110 (96%)  4 (4%)  85.5  (85–95) | n (% of 47)  40 (85%)  7 (15%)  85.0  (85–113) |

Abbreviations: CI, confidence interval; CLL, chronic lymphocytic leukaemia; DLBCL, diffuse large B-cell lymphoma; FL, follicular lymphoma.
